# Impact of polishing system on surface roughness of different ceramic surfaces after various pretreatments and bracket debonding

**DOI:** 10.1007/s00784-023-05058-3

**Published:** 2023-05-11

**Authors:** Rebecca Jungbauer, Anja Liebermann, Christian M. Hammer, Daniel Edelhoff, Peter Proff, Bogna Stawarczyk

**Affiliations:** 1 Department of Orthodontics, University Medical Centre Regensburg, Franz-Josef-Strauß-Allee 11, 93053 Regensburg, Germany; 2 Department of Prosthetic Dentistry, University Hospital, LMU Munich, Munich, Germany; 3 Department of Prosthetic Dentistry, Faculty of Medicine and University Hospital Cologne, University of Cologne, Cologne, Germany; 4 Institute of Functional and Clinical Anatomy, Friedrich Alexander University Erlangen-Nürnberg, Erlangen, Germany; 5 Faculty of Science and Medicine, University of Fribourg, Anatomy Unit, Fribourg, Switzerland

**Keywords:** Surface roughness, Pretreatment, Bracket debonding, Polishing, Ceramics

## Abstract

**Objective:**

Evaluating various polishing methods after bracket debonding and excessive attachment material removal for different ceramics and pretreatments.

**Material and methods:**

Zirconia (ZrO2), leucite (LEU) and lithium disilicate (LiSi) specimens were pretreated with a) silica coated alumina particles (CoJet); LEU and LiSi additionally with b) hydrofluoric acid (HF), c) Monobond Etch&Prime (MEP), d) silicium carbide grinder (SiC) before bracket bonding, shearing off, ARI evaluation, excessive attachment material removal and polishing with i) Sof-Lex Discs (Soflex), ii) polishing paste (Paste), iii) polishing set (Set). Before/after polishing surface roughness (Ra) was measured with a profilometer. Martens hardness parameter were also assessed.

**Results:**

Irrespective of pretreatment Ra of LEU increased the most, followed by LiSi and ZrO2 (*p* < 0.001, SiC: *p* = 0.012), in accordance with the measured Martens hardness parameter. CoJet/SiC caused greater roughness as HF/MEP (*p* < 0.001). The ZrO2 surface was rougher after polishing with Paste/Set (*p* < 0.001; *p* = 0.047). Ra improved in the LEU/CoJet, LEU/SiC and LiSi/SiC groups with Soflex/Set (*p* < 0.001), in the LiSi/CoJet and LEU/HF groups by Soflex (*p* = 0.003, *p* < 0.001) and worsened by Paste (*p* = 0.017, *p* < 0.001). Polishing of HF or MEP pretreated LiSi with Set increased Ra (*p* = 0.001, *p* < 0.001), so did Paste in the LEU/MEP group (*p* < 0.001).

**Conclusions:**

Paste couldn’t improve the surfaces. Soflex was the only method decreasing Ra on rough surfaces and not causing roughness worsening. Polishing of LEU/LiSi after MEP, LEU after HF pretreatment doesn´t seem to have any benefit.

**Clinical Relevance:**

To avoid long-term damage to ceramic restorations, special attention should be paid to the polishing method after orthodontic treatment.

**Supplementary Information:**

The online version contains supplementary material available at 10.1007/s00784-023-05058-3.

## Introduction

In recent years, the number of adults wishing for orthodontic correction has been increasing due to a wide offer of less visible or invisible treatment options such as aligners or lingual braces [1]. For most treatments, it is necessary to bond brackets or attachments to natural teeth surfaces. In case of treating adult patients there must be a reliable bonding protocol for bonding to different kinds of restoration surfaces, in many cases ceramics such as monolithic zirconia (ZrO2), leucite (LEU) or lithium silicate ceramics (LiSi) [2–4]. Every bracket loss prolongs treatment time [5] and must therefore be kept to a minimum. There are different pretreatment protocols in the literature for the different types of ceramic, which are all based on the principle to roughen the surface to create mechanical retention for the orthodontic attachment material. Depending on the ceramic type the surface can be roughened mechanically by air-abrasion or using grinders, chemically with hydrofluoric acid (HF) or ammonium polyfluoride (Monobond Etch&Prime, MEP) or using a combination of mechanical and chemical pretreatment (tribochemical silicia coating, CoJet) [4, 6]. Usually, in accordance with recommendations for prosthetic purposes the roughening is followed by the application of a silane, adhesive or universal adhesive, again depending on the type of ceramic [7, 8].

Apart from a reliable bonding of the brackets throughout the treatment, the surface must not be damaged by roughening the surface, taking off the bracket or removing the excessive attachment material. Furthermore, the surface roughness (microcracks) of the ceramic has an impact on the esthetics but also on physical properties [9]. A smoothly polished surface is important for long-term clinical success reducing the risk of chipping (propagation of the microcracks) but also plaque adhesion [10–12]. Therefore, after debonding the surface must be polished by the orthodontist very carefully. For intraoral polishing different tools are available for example ceramic polishing kits, alumina coated discs (Soflex) or polishing pastes that can be applied with a brush or a rubber cup. In the literature, there are some studies investigating polishing of ceramic surfaces after orthodontic treatments [6, 13–15]. But a general recommendation can still not be derived. To the best of the authors’ knowledge, none of the available studies have investigated the influence of different ceramics including ZrO2 and various pretreatment methods and different tools for polishing.

Therefore, the aim of this investigation was to analyze the impact of different pretreatment methods on surface morphology and compare various polishing methods for different types of ceramic after bracket debonding. The null hypothesis investigated whether all polishing methods show similar outcome, regarding roughness irrespective of the ceramic and pretreatment. The second null hypothesis assumed no impact of pretreatment method on surface roughness after bracket debonding.

## Materials and methods

### Specimen preparation

Forty-five specimens with the dimension of 5 × 5 × 3 mm were cut out of Ceramill Zolid FX Multilayer (ZrO2, Amann Girrbach, Koblach, Austria) zirconia blanks and sintered according to the manufacturer’s recommendations in the sintering furnace (LHT 02/16, Nabertherm, Lilienthal/Bremen, Germany). To fabricate the 180 leucite specimens (LEU) a layering technique and a silicone mold to standardize the form were used. To compensate for the shrinkage of the sintering process dentin powder was added for the second firing (Austromat 654, preee-i-dent, Dekema, Freilassing, Germany). The slurry was condensed into the mold with a vibrator at 50 Hz for 2 s (ElektroVibrator Porex, Renfert, Hilzingen, Germany). Squared specimens (n = 180) of 3 mm thickness were cut from lithium disilicate blanks (LiSi, IPS e.max CAD A2/C14, Ivoclar, Schaan, Liechtenstein) and underwent a specific treatment for final crystallization (program IPS e.max CAD Crystal/Glaze HT/LT, furnace: Programat EP 5000, Ivoclar). All 405 specimens were embedded in the chemically curing resin ScandiQuick (A and B; Scan-Dia, Hagen, Germany).

Finally, they were subsequently polished up to P1200 (ZrO2) or P2000 (LiSi, LEU) (SiC-Paper, Struers, Ballerup, Denmark) in an automatic polishing device (20 s, Tegramin 20, Struers) under permeant water cooling and stored in distilled water at room temperature (23 °C) until further usage.

### Pretreatment and bonding procedure

Before further treatment all specimens were cleaned with a pumice/water mixture (40:50 g) and a polishing brush (Busch & Co, Engelskirchen, Germany) from left to right and up and down for 3 s each at a speed of 3.000 rpm. Each type of ceramic was then pretreated according to Table 1. Directly after bonding, all specimens were stored in distilled water. Five specimens per group were stored at 37° C for 24 h, five underwent 500 thermal cycles (5°/55° C), dwell time 2 s and five were stored at 37° C for 90 days.

**Table 1:** Study workflow presenting different pretreatments of ceramics used

Ceramic	Pretreatment	Bracket bonding
ZrO2	CoJet* (3 M, Monrovia, USA): 2 s at 90°, 10 mm distance, 2 bars, rinsing, air-drying + CF (Clearfil Ceramic Primer Plus, Kuraray Noritake, Okayama, Japan): application and air-drying	Clarity Advanced bracket (3 M) bonded with a thin layer of Transbond XT Adhesive (3 M), slight pressure, excess removal, 3 s light curing trough the bracket (1600 mW/cm^2^, Ortholoux luminous curing light, 3 M)
LEU	4% Porc-Etch (HF, Reliance Orthodontic Products, Itaca, USA): 60 s, rinsing, air-drying + Porcelain Conditioner (Reliance Orthodontic Products): 60 s	
	Monobond Etch&Prime (MEP, Ivoclar, Schaan, Liechtenstein): 20 s agitating, 40 s allowing to react, rinsing, air-drying	
	CoJet* (3 M): 2 s at 90°, 10 mm distance, 2 bars, rinsing, air-drying + RelyX Ceramic Primer (RXP, 3 M): 60 s	
	Silicium carbid grinder (SiC, VOCO, Cuxhaven, Germany): 10 × horizontally and vertically, 10.000 rpm + Cimara Silan (VOCO): 120 s	
LiSi	4% Porc-Etch (HF Reliance Orthodontic Products): 20 s, rinsing, air-drying + Porcelain Conditioner (Reliance Orthodontic Products): 60 s	
	Monobond Etch&Prime (MEP, Ivoclar): 20 s agitating, 40 s allowing to react, rinsing, air-drying	
	CoJet* (3 M): 2 s at 90°, 10 mm distance, 2 bars, rinsing, air-drying + RelyX Ceramic Primer (RXP, 3 M): 60 s	
	Silicium carbid grinder (SiC, VOCO): 10 × horizontally and vertically, 10.000 rpm + Cimara Silan (VOCO): 120 s	

^*^30 μm silica coated alumina powder, ZiO2: zirconia, LEU: leucite, LiSi: lithium silicate

### Debonding procedure

To ensure a standardized deboning, the specimens were dried and stored at room temperature (23 °C) for 1 h. They were placed in a special test apparatus in the universal testing machine (RetroLine, Zwick/Roell, Ulm, Germany) and a parallel to the ceramic substrate acting compressive force was applied in an occluso-gingival direction at a crosshead speed of 1 mm/min until debonding. Afterwards, the adhesive remnant index (ARI) was evaluated for each specimen using a microscope with 10 × magnification (Bresser, Rhede, Germany) as follows [16]: 0 = no remaining attachment material (AM) on the ceramic, 1 = less than 50% remaining on the ceramic, 2 = more than 50% remaining on the ceramic, and 3 = 100% AM remaining on the ceramic.

The remaining attachment material was then completely removed with a tungsten bur (Busch, Engelskirchen, Germany) at 10.000 rpm without pressure and no water cooling. The bur was changed after every 5 specimens.

### Polishing methods

The specimens were randomly divided into 3 groups (n = 15) within each pretreatment and ceramic and polished with:

1. Sof-Lex fin and polish Disks (Soflex): thin coarse, thin medium, thin fine, and superfine at 10.000 rpm using the mandrel and a handpiece. With each disk the surface was wet polished under constant movement and no pressure for 20 s. After polishing 5 specimens, the Sof-Lex disks were changed.
2. A ceramic polishing set (Set, pre, fine, high shine, REF 4313B, Komet Dental, Lemgo, Germany) under water cooling at 6.000 rpm under constant movement, no pressure and for 20 s each. The same polishing set was used for the whole group.
3. A polishing rubber cup (Cup, Pro-Cup light blue, Kerr, Rastatt, Germany) and DirectDia Paste diamond polishing paste (Paste, DirectDia Paste, Shofu, Kyoto, Japan) at 3.000 rpm, wet, for 60 s and under constant movement and no pressure. The same rubber cup was used for one entire group.

### Surface measurements

A profilometer (S6P, Mahr, Göttingen, Germany) was used for the quantitative surface characterization. The surface topography was measured before pretreatment, after excessive attachment material removal with the tungsten bur and after final polishing. The area where the bracket was bonded before was marked before measurements, that were all taken within this area. The mean of six orthogonal measurements of the roughness average (Ra) was recorded.

### Scanning electron microscopy (SEM)

From every ceramic type (ZrO2, LEU, LiSi) those two specimens with the highest and lowest Ra values after polishing were chosen for SEM (n = 6). Additionally, the polishing paste was spread on an aluminum stub and dried for 7 days at 80° C. The surfaces were sputter-coated with a 20 nm layer of gold with the Leica EM ACE200 system (Leica Mikrosysteme, Vienna, Austria) and viewed using a JEOL scanning electron microscope (JSM-IT 300LV, JEOL, Eching, Germany) at 30 × and 250 × magnification.

### Martens hardness parameter

To analyze the Martens hardness parameter (HM in N/mm^2^ and EIT in kN/mm^2^), a universal hardness testing machine (ZHU 0.2/Z2.5, Zwick Roell, Ulm, Germany) was used. Therefore, one specimen of each substrate (ZrO2, LEU, LiSi) was measured four times. Therefore, the diamond indenter pyramid (α = 136°) of the testing machine was pressed vertically into specimen surface with a load of 9.81 N for 10 s. The maximum depth of the indenter in surface was 0.01 mm. Martens hardness (HM) and indentation modulus (EIT) values were calculated (testXpert V12.3 Master, Zwick).

### Statistical method

For statistical analyses the software IBM SPSS Statistics V.28 (IBM, Armonk, NY, USA) was used. ΔRa were calculated by subtracting the value after polishing from the value before polishing. The influence of ceramic type, pretreatment, and polishing method was analyzed with a global univariate ANOVA test with partial eta-squared ${\upeta }_{p}^{2}$. The Shapiro–Wilk test indicated a violation of normal distribution of more than 5% of the data. For the comparison of Ra values before and after polishing (effectiveness of polishing, within each group) the Wilcoxon-test was used. For analyzing the influence of polishing methods and pretreatment on the polishing result (change of Ra; between groups) and the influence of pretreatment before polishing Kruskal–Wallis-tests followed by Dunn-Bonferroni post-hoc tests were applied. For the pairwise comparison the Bonferroni correction of the p-values was utilized. *P* < 0.05 was considered as statistically significant.

## Results

Prior to pretreatment, the initial Ra value of LiSi was measured with 0.032 µm (IQR: 0.010 µm), of LEU with 0.054 µm (IQR: 0.016 µm), and of ZrO2 with 0.071 µm (IQR: 0.016 µm). The Ra measurements after polishing were more influenced by the pretreatment (${\eta }_{p}^{2}$=0.709, *p* < 0.001), followed by the ceramic (${\eta }_{p}^{2}$=0.703, *p* < 0.001), and the method of polishing (${\eta }_{p}^{2}$=0.570, *p* < 0.001).

### Impact of the different pretreatments before polishing

**Fig. 1:**
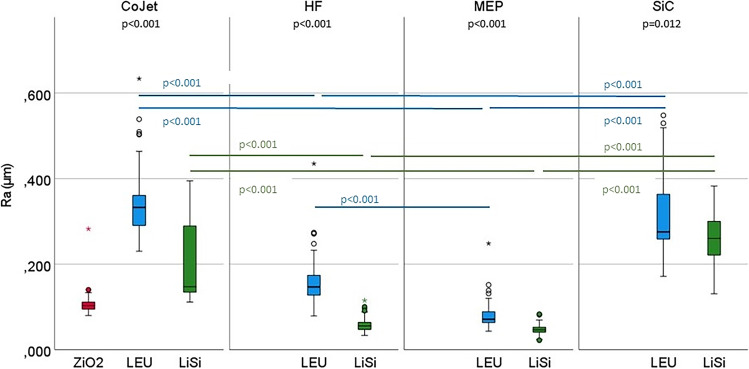
Ra values after pretreatment of ceramics and removal of excessive attachment material. Upper p-values (black) indicate differences of Ra values after one pretreatment between ceramic types, p-values within the graphic indicate differences regarding Ra values after different pretreatments of one ceramic type (blue: LEU, green: LiSi), Kruskal–Wallis-tests followed by Dunn-Bonferroni post-hoc tests were applied (*p* < 0.05); Ra: roughness average, HF: hydrofluoric acid, MEP: Monobond Etch&Prime, SiC: silicium carbid grinder, Paste: polishing paste, Set: polishing set, ZiO2: zirconia, LEU: leucite, LiSi: lithium silicate

With regards to the different ceramic surfaces, after pretreatment with silica coated alumina powder (CoJet), Ra values of ZrO2 were lower compared to LiSi (*p* < 0.001), and LEU (*p* < 0.001). Ra values of LiSi were found to be lower than those of LEU (*p* < 0.001). Ra of LEU was rougher than of LiSi after the use of HF, MEP, and SiC (HF: *p* < 0.001, MEP: *p* < 0.001, SiC: *p* = 0.012) (Fig. 1).

Comparing the various pretreatments within the LEU and the LiSi group, the different treatment methods overall yielded different Ra values (*p* < 0.001). The highest values were caused by CoJet and SiC, those of HF were lower (CoJet/HF and SiC/HF: *p* < 0.001), followed by the lowest after MEP pretreatment (CoJet/MEP, SiC/MEP: *p* < 0.001). The difference between HF and MEP was significant (*p* < 0.001) in the LEU, not in the LiSi group considering the Ra values (Fig. 1).

### Result of different polishing methods within each ceramic and pretreatment group

The Ra values before and after polishing within each ceramic and pretreatment group were analyzed. Polishing with Paste and Set resulted in higher Ra values of the CoJet pretreated ZrO2 surface as before polishing (Paste: *p* < 0.001; Set: *p* = 0.047). Within the LEU/CoJet group, Soflex and Set polishing improved the surface (both: *p* < 0.001), Paste deteriorated the surface smoothness (*p* = 0.009). The HF pretreated LEU was smoother using Soflex (*p* < 0.001) and rougher with Paste (*p* < 0.001). The LEU surface after MEP pretreatment remained rougher after Paste polishing as before (*p* < 0.001). Within the LEU/SiC group, Soflex and Set polishing left a smoother surface (both *p* < 0.001), Paste a rougher one (*p* = 0.008). The LiSi surface after CoJet pretreatment was improved using Soflex (*p* = 0.003) and was rougher using Paste (*p* = 0.017). Set polishing worsened LiSi after HF as well as after MEP pretreatment (HF: *p* = 0.011; MEP: *p* < 0.001). The SiC pretreated LiSi surface was improved by Soflex and Set polishing (both *p* < 0.001) (Table 2). Recommendations for each ceramic/pretreatment combination based on the Ra values before and after polishing are summarized in supplementary Fig. 1.

**Table 2:** Medians (MD) and interquartile ranges (IQR) of Ra values before and after polishing in μm

		Soflex				Paste				Set			
		Ra before polishing		Ra after polishing		Ra before polishing		Ra after polishing		Ra before polishing		Ra after polishing	
Ceramic	Pretreatment	MD (IQR)	Min/ Max	MD (IQR)	Min/ Max	MD (IQR)	Min/ Max	MD (IQR)	Min/ Max	MD (IQR)	Min/ Max	MD (IQR)	Min/ Max
ZiO2	CoJet	0.102^a^ (0.012)	0.086/ 0.283	0.108^a^ (0.014)	0.089/ 0.131	0.110^b^ (0.020)	0.088/ 0.140	0.148^c^ (0.013)	0.117/ 0.165	0.095^d^ (0.013)	0.080/ 0.126	0.105^e^ (0.019)	0.082/ 0.141
LEU	CoJet	0.294^a^ (0.098)	0.230/ 0.505	0.113^b^ (0.026)	0.086/ 0.183	0.341^c^ (0.133)	0.238/ 0.539	0.404^d^ (0.080)	0.326/ 0.509	0.332^e^ (0.040)	0.271/ 0.634	0.215^f^ (0.022)	0.194/ 0.260
	HF	0.149^a^ (0.029)	0.079/ 0.184	0.101^b^ (0.037)	0.070/ 0.130	0.143^c^ (0.058)	0.119/ 0.274	0.217^d^ (0.067)	0.152/ 0.314	0.147^e^ (0.128)	0.111/ 0.435	0.201^e^ (0.024)	0.164/ 0.223
	MEP	0.066^a^ (0.027)	0.047/ 0.132	0.081^a^ (0.019)	0.068/ 0.107	0.075^b^ (0.037)	0.043/ 0.152	0.072^b^ (0.026)	0.056/ 0.139	0.074^c^ (0.034)	0.050/ 0.248	0.196^d^ (0.013)	0.168/ 0.226
	SiC	0.319^a^ (0.120)	0.221/ 0.548	0.114^b^ (0.029)	0.093/ 0.178	0.269^c^ (0.043)	0.172/ 0.351	0.322^d^ (0.040)	0.161/ 0.423	0.261^e^ (0.223)	0.223/ 0.530	0.189^f^ (0.031)	0.164/ 0.210
LiSi	CoJet	0.147^a^ (0.155)	0.124/ 0.354	0.123^b^ (0.049)	0.074/ 0.165	0.137^c^ (0.125)	0.111/ 0.395	0.250^d^ (0.041)	0.122/ 0.336	0.148^e^ (0.188)	0.120/ 0.376	0.154^e^ (0.013)	0.123/ 0.168
	HF	0.052^a^ (0.017)	0.033/ 0.088	0.049^a^ (0.011)	0.042/ 0.064	0.053^b^ (0.018)	0.042/ 0.115	0.053^b^ (0.013)	0.037/ 0.186	0.059^c^ (0.035)	0.047/ 0.100	0.084^d^ (0.026)	0.054/ 0.158
	MEP	0.052^a^ (0.015)	0.033/ 0.082	0.053^a^ (0.015)	0.040/ 0.070	0.045^b^ (0.017)	0.027/ 0.069	0.039^b^ (0.008)	0.030/ 0.047	0.045^c^ (0.011)	0.022/ 0.083	0.127^d^ (0.025)	0.071/ 0.150
	SiC	0.285^a^ (0.095)	0.141/ 0.383	0.096^b^ (0.033)	0.061/ 0.145	0.245^c^ (0.098)	0.143/ 0.371	0.237^c^ (0.059)	0.174/ 0.351	0.269^d^ (0.064)	0.131/ 0.349	0.095^e^ (0.029)	0.053/ 0.147

Different ceramics and pretreatments are shown in the rows, polishing methods are represented in the columns. Statistically significant differences (*p* ≤ 0.05) are specified by superscript letters. Different letters in the line indicate significant differences before and after polishing within one ceramic/polishing/pretreatment group. Values sharing the same letter have no difference. For statistical comparison the Wilcoxon-test was used. Ra: roughness average, HF: hydrofluoric acid, MEP: Monobond Etch&Prime, SiC: silicium carbid grinder, Paste: polishing paste, Set: polishing set, ZiO2: zirconia, LEU: leucite, LiSi: lithium silicate

### Comparison between polishing methods within the different ceramic and pretreatment groups

Positive values of ΔRa indicate an improvement in terms of less surface roughness and negative values a worsened (rougher) surface. The Ra changes after the three different polishing methods within each combination of ceramic and pretreatment were compared. Within the ZrO2/CoJet there was a greater surface change (rougher) after Paste polishing (-0.035 µm) compared to Set (-0.017 µm, *p* = 0.015) and Soflex (-0.002 µm; *p* < 0.001).

**Fig. 2:**
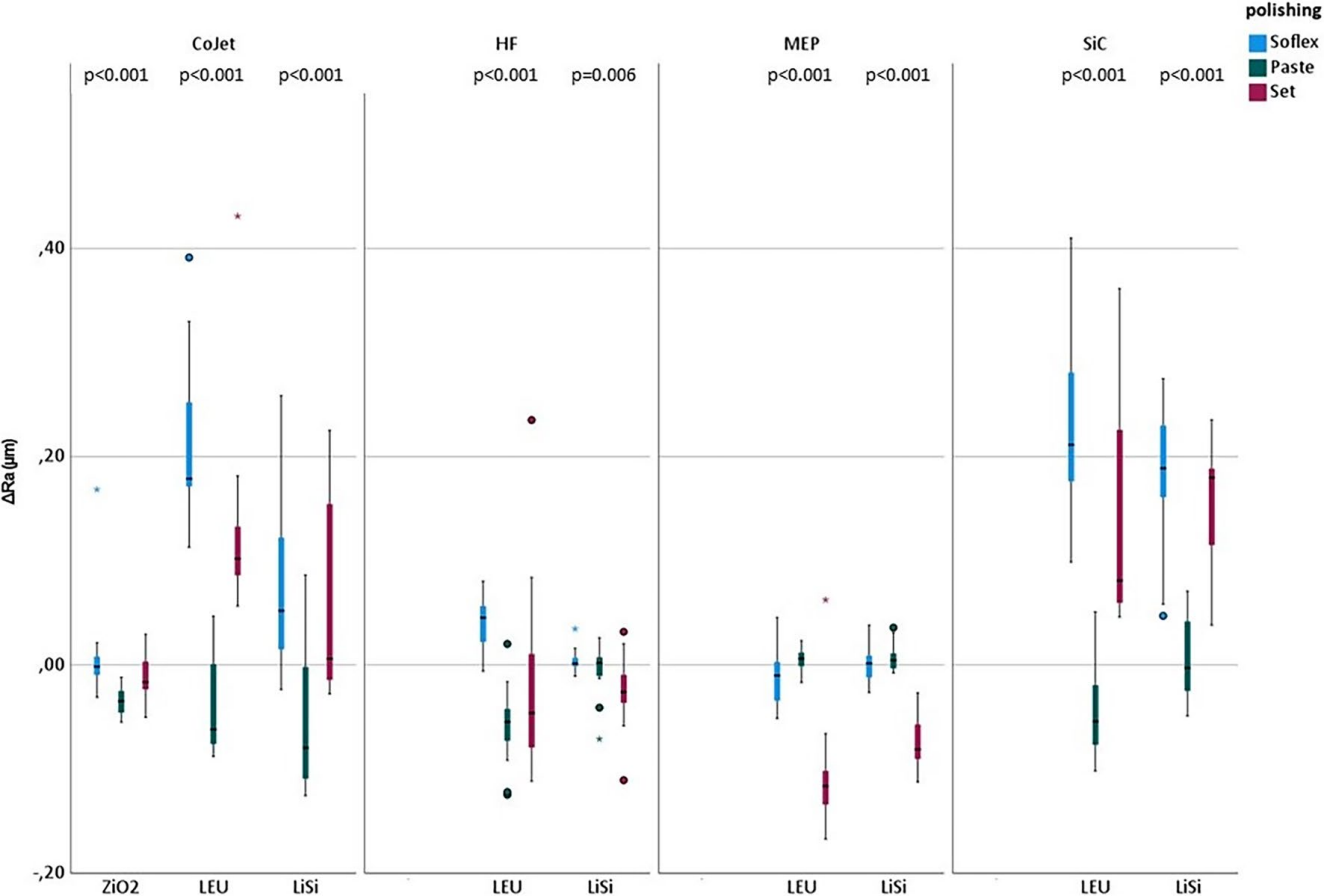
Change of Ra values depending on pretreatment, ceramic and polishing. The boxplots show the surface change (Ra value) after polishing in comparison to before. Positive values indicate a smoother, negative a rougher surface. As global statistical test the Kruskal–Wallis-test was used. P-values are given in the figure, post hoc p-values (pairwise comparison) are described in the main text. Ra: roughness average, HF: hydrofluoric acid, MEP: Monobond Etch&Prime, SiC: silicium carbid grinder, Paste: polishing paste, Set: polishing set, ZiO2: zirconia, LEU:leucite, Lisi: lithium silicate

After pretreatment with CoJet the LEU surface was more influenced by Soflex (0.179 µm) and Set (0.102 µm) than by Paste (-0.062 µm; Soflex: *p* < 0.001; Set: *p* = 0.001). Soflex polishing (0.045 µm) influenced the surface less than Paste (-0.055 µm, *p* < 0.001) and Set (-0.046 µm, *p* = 0.007) in the LEU/HF group. Set had a greater impact after MEP (-0.116 µm, *p* = 0.180) pretreatment of LEU as Soflex (-0.010 µm, *p* = 0.004) and Paste (-0.062 µm, -0.003 µm). In the LEU/SiC group there was a greater change of the Ra values using Soflex (0.211 µm, *p* < 0.001) and Set (0.081 µm, *p* = 0.001) compared to Paste (-0.054 µm) (Fig. 2).

The CoJet pretreated LiSi surface was more influenced by Paste polishing (-0.081 µm) as by Soflex (0.052 µm; *p* = 0.001) and Set (0.0006 µm; *p* = 0.016). In the HF/LiSi group Set (-0.026 µm) caused more surface changes than Soflex (0.001 µm, *p* = 0.006). Set had a greater impact after MEP (0.180 µm) pretreatment of LiSi as Soflex (0.002 µm, *p* < 0.001) and Paste (-0.003 µm; *p* < 0.001). In the LiSi/SiC group there was a greater change of the Ra values using Soflex (0.189 µm, *p* < 0.001) and Set (0.180 µm; *p* < 0.001) compared to Paste (-0.003 µm) (Fig. 2).

### Adhesive remnant index (ARI)

After pretreatment with CoJet and HF higher ARI scores (2–3) were detected more frequently within all ceramics. An ARI of 0–1 was more frequent after MEP pretreatment of LiSi and LEU. SiC pretreatment resulted more often in higher ARI (2–3) in the LEU group and in lower ARI scores (0–1) in the LiSi group (Table 3).

**Table 3:** Distribution of ARI scores

Ceramic	Pretreatment	ARI				Total
		0	1	2	3	
ZrO2	CoJet	0 (0.0%)	15 (33.3%)	26 (57,8%)	4 (8.9%)	45 (100%)
LEU	CoJet	0 (0.0%)	1 (2.2%)	5 (11.1%)	39 (86,7%)	45 (100%)
	HF	0 (0.0%)	1 (2.2%)	1 (2.2%)	43 (95.6%)	45 (100%)
	MEP	19 (42.2%)	15 (33.3%)	4 (8.9%)	7 (15.6%)	45 (100%)
	SiC	0 (0.0%)	2 (4.4%)	10 (22.2%)	33 (73.3%)	45 (100%)
LiSi	CoJet	3 (6.7%)	13 (28.9%)	6 (13.3%)	23 (51.1%)	45 (100%)
	HF	0 (0.0%)	2 (4.5%)	8 (18.2%)	34 (77.3%)	45 (100%)
	MEP	15 (33.3%)	14 (31.1%)	2 (4.4%)	14 (31.1%)	45 (100%)
	SiC	23 (51.1%)	12 (26.7%)	3 (6.7%)	7 (15.6%)	45 (100%)

Number and percentage of specimen and rated ARI score (0–3)

Data are given for every surface and respective pretreatment separately. HF: hydrofluoric acid, MEP: Monobond Etch&Prime, SiC: silicium carbid grinder, ZiO2: zirconia, LEU:leucite, LiSi: lithium silicate

### Martens hardness parameter

ZrO2 showed the highest median values of the Martens hardness parameters Martens hardness (HM) and indentation modulus (EIT) HM: MD = 8397 N/mm^2^, IQR = 4152 N/mm^2^; EIT: MD = 173 N/mm^2^, IQR = 107 N/mm^2^), followed by LiSi (MH: MD = 3853 N/mm^2^, IQR = 315 N/mm^2^; EIT: MD = 77 N/mm^2^, IQR: 10.5 N/mm^2^). LEU presented the lowest values (HM: MD = 2716 N/mm^2^, IQR = 759 N/mm^2^; EIT: MD = 48 N/mm^2^, IQR = 10.5 N/mm^2^).

### Scanning electron microscopy (SEM)

**Fig. 3:**
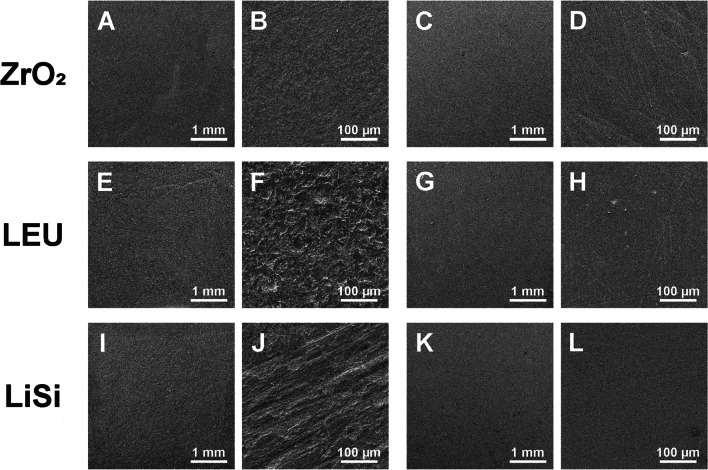
Scanning electronic micrographs of former bonding area after bracket and attachment material removal as well as final polishing. Each row represents the different types of ceramic. In the columns two different magnifications of a combination of pretreatment and polishing method are shown: A/B: CoJet/Paste, C/D: CoJet/Set, E/F: CoJet/Paste, G/H: MEP/Paste, I/J: SiC/Paste, K/L: MEP/Paste. HF: hydrofluoric acid, MEP: Monobond Etch&Prime, SiC: silicium carbid grinder, Paste: polishing paste, Set: polishing set, ZiO2: zirconia, LEU:leucite, Lisi: lithium silicate

**Fig. 4:**
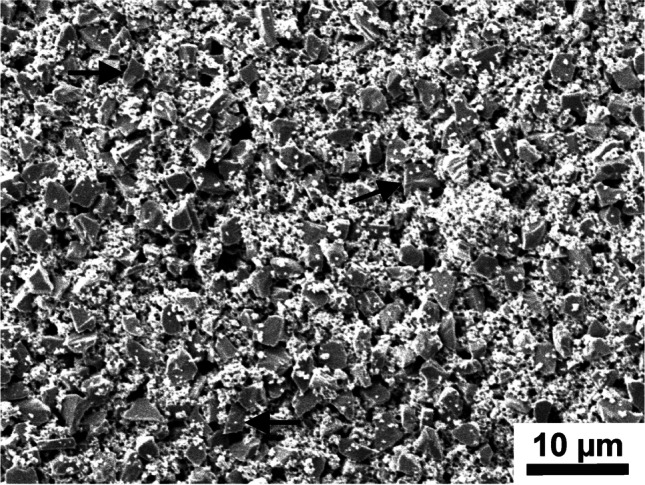
Scanning electronic micrograph of dried polishing paste. Sharp ceramic particles are discernible – examples are indicated by the arrows

Scanning electronmicrographs of the ceramic surface after final polishing are presented in Fig. 3 and of the Paste in Fig. 4. LEU and LiSi presented a distinctly irregular surface with partly visible streaks and minor structural defects. On the other hand, ZrO2 specimens have more regular, finer surface structures. The polishing paste revealed inorganic components (ceramic fillers) with pointed particles.

## Discussion

After bracket debonding from ceramic surfaces there is no general recommendation or standard operating procedure of how to polish the surface properly. Furthermore, different pretreatment methods might require different polishing methods which might also depend on the ceramic type of restoration. Therefore, the aim of this investigation was to compare different polishing methods after bracket debonding, applied to different ceramic surfaces after various pretreatments.

In the literature, Ra values less than 0.2 μm are recommended to reduce bacterial and plaque adhesion [17, 18]. After Soflex polishing, all Ra values were below the required 0.2 μm, irrespective of the ceramic type. Polishing with Paste resulted in higher values (> 0.2 μm) in the following groups: LEU combined with CoJet, HF, and SiC as well as LiSi pretreated with CoJet and SiC. The LEU surface could also not be smoothened below 0.2 μm after any of the tested pretreatments by using SET apart from the SiC pretreated specimens.

The null hypothesis (“all polishing methods show similar outcome, regarding roughness irrespective of the ceramic and pretreatment”) was rejected. Soflex polishing improved the surface in the LEU group after CoJet, HF, and SiC pretreatment and in the LiSi group after CoJet and SiC pretreatment. Therefore, it can be concluded that Soflex improved rough surfaces and did not damage the smoother surfaces. Karan et al. also found Soflex polishing to be most effective in comparison to polishing wheels and a polishing paste after pretreatment of different silicate ceramics with sandblasting and HF etching followed by bracket debonding [6]. In accordance with the present investigation, the effectiveness of Soflex polishing for ceramic polishing has been previously described in the literature [18–21].

Polishing with Paste couldn´t improve the surface roughness in any of the tested groups. Irrespective of the ceramic type, polishing with Paste after CoJet pretreatment resulted in higher Ra values compared to unpolished CoJet surfaces. In the LEU group, the same was true for HF and SiC pretreatment. This implies that Paste polishing worsened surfaces with already high Ra values (after CoJet in general, SiC, and HF of LEU surfaces), whereas no impact on smoother surfaces as for example after MEP pretreatment was analyzed. This finding is supported by the SEM images where after CoJet pretreatment of LEU a very rough surface after Paste polishing remained as well as for SiC pretreated LiSi (Fig. 3F/J). In contrast after Paste polishing of the smooth surfaces (LEU and LiSi after MEP pretreatment), the surface remained smooth (Fig. 3H/L). A previous investigation found very high Ra values after polishing of leucite ceramic specimens with the same Paste that were above the clinical acceptable value [22], confirming the present results. In accordance with other investigations, they recommend a Paste polishing only in combination with a polishing kit as final procedure [22–24], which was not tested in the present study. It can be assumed that especially softer ceramics (here LEU) suffer more from this specific Paste polishing if the surface is very rough as for example after CoJet and SiC pretreatment.

After pretreating the LEU surface with CoJet and the LEU and LiSi surfaces with SiC, roughness was improved by Set polishing. In contrast, the surface was rougher as before polishing after CoJet pretreatment of ZrO2, MEP on LEU and LiSi as well as HF on the LEU surface. Set is a polishing set for ceramic restorations including polisher for pre-polishing, polishing and high-gloss polishing. The SEM picture of the Set polished ZrO2 after CoJet pretreatment shows a very scratched (Fig. 3D) but not porous surface compared to the Paste polishing (Fig. 3B). It seems that Set improves rougher surfaces but can damage smoother surfaces, which might be due to the stiffness of the polishing devices, which are interspersed with ceramic particles and could therefore be more influenced by pressure during use. In comparison, Soflex is very flexible and possibly less susceptible to increased pressure. Two further investigations concluded that using the same Set reducing the Ra values after the imitation of intraoral adjustments on a LiSi surface below the critical threshold of 0.2 μm was not possible [25], neither was an adequate polishing of ZiO2 and LEU after removal of the glaze layers [26]. A comparison with other investigations using different polishing kits is difficult and further research is desirable here.

It generally needs to be questioned, if polishing after the use of MEP on LiSi and LEU, and HF on LiSi surfaces is required or not. The Ra values were very low in these groups after removal of excessive attachment material with a tungsten carbide bur and could not be improved by any of the polishing methods but worsened by some.

Comparing the impact of surface changes of the different polishing methods within the ceramic and pretreatment groups, interestingly polishing with Paste damaged the ZrO2 surface more than the other polishing methods. This finding is supported by the SEM pictures, where the whole ZrO2 surface was generally rough after Paste polishing (Fig. 3B), and after Set there are smoother parts visible with some scratches (Fig. 3D). The Paste polishing also had a greater negative influence in the CoJet/LiSi group as Soflex and Set had a positive impact. After HF application on LEU, Set and Paste had a more negative impact on the surface roughness than Soflex had a positive one. It seems that Paste has a greater negative influence on rougher surfaces which might be explained by the fact, that the sharp-edged ceramic particles, that were visible in the SEM picture (Fig. 4), have a larger target area on rougher surfaces to cause even more surface roughness. HF etching of silicate ceramics results in a surface that is porous and has microcavities caused by the dissolving of the glass phase [27]. Considering the HF pretreated LEU surface, the more negative influence of Paste might be due to this porous surface texture allowing further breaking out leucite crystals with the sharp-edged ceramic particles. Possibly, further polishing with different polishing pastes might have improved the surface values. Liebermann et al. found significant differences between prophylactic polishing pastes on surface roughness of CAD/CAM composites, where some of them also caused more abrasions on the artificial surfaces [28]. In general, it needs to be questioned, if multi-step systems could be of advantage in everyday clinical practice. Further research is necessary here.

The CoJet and SiC pretreated LEU surface roughness could be more positively changed with Soflex and Set as negatively by Paste. Soflex and Set had greater positive impact on the SiC pretreated LiSi than Paste. These three groups were the ones with the highest Ra values after pretreatment. It seems that the positive influence of Soflex and SiC polishing also increases with the surface roughness.

Set polishing had the greatest (negative) influence on the MEP pretreated LEU. Within the HF/LiSi group, Set also had the greatest impact (negative) on the surface. After MEP pretreatment of LiSi the polishing with Set caused greater roughness than Soflex and Paste could smoothen the surface. In contrast to Paste it seems that Set has especially a negative impact on rather smooth surfaces, which might be because the polishing cups are rather stiff in comparison the rubber cups and the abrasive particles on the shaft might exert a very high abrasive pressure.

The second null hypothesis had to be rejected as well (“no impact of pretreatment method on surface roughness, and depth after bracket debonding”). The CoJet pretreatment of the ceramics caused higher Ra values of the LEU surface followed by LiSi and had the least impact on ZrO2. This phenomenon might be explained by the hardness of the substrate. The softer the substrate material, the more susceptible it is to air-abrading with particles at a pressure of 2 bar. The other pretreatment methods (HF, MEP, SiC) also had a higher influence on the LEU than LiSi surface. LEU ceramics are not only softer, but also have lower mechanical properties. The abrasion resistance of LEU ceramics is also lower than that of LiSi ceramics. This also explains the higher Ra values in this group. This is in accordance with the measured HM and EIT values of the three different ceramic types, where ZrO2 presented the highest values, followed by LiSi and LEU with the lowest values. Comparing the influence of the pretreatment methods on the surface with each other the use of CoJet as well as SiC resulted in the highest Ra, followed by both HF and MEP, with exception of the Ra values on LEU where MEP caused lower values. Herion et al. also found higher Ra values after CoJet than after phosphoric acid pretreatment of a LEU ceramic before bracket bonding [13]. MEP only causes minor surface roughness changes as the surface is coated in one step [27]. The fact that generally mechanical/tribochemical pretreatment causes greater roughness is in accordance with the literature [29]. When using this kind of pretreatment, the clinician needs to be aware of higher requirements for polishing after debonding.

After both pretreatments, CoJet and HF, irrespective of the ceramic, and SiC pretreatment of LEU resulted in a higher ARI than after MEP or SiC pretreatment of LiSi. A high ARI means that more attachment material remains on the specimen than on the bracket base after taking off the bracket. Therefore, the adhesion between ceramic specimens and attachment material seems to be similar although CoJet caused rougher surfaces than HF for example. From a clinical perspective, this implies that rougher surfaces do not necessarily cause better adhesion for the bonding than chemical pretreatments which is in accordance with studies evaluating shear bond strength after different pretreatment methods [4].

Apart from Ra values, Rz values were also investigated but led to the same results as those of the Ra values. To avoid redundancy, the authors decided to report only the Ra values. No further profile measurements were performed, which can be considered as limitation but is in accordance with comparable studies [6, 18, 22, 30]. Due to its in-vitro nature this investigation has some limitations to consider. Other authors removed the bracket with a plier similar to intraoral debonding [13]. Although the classical shear bond strength testing by using a universal testing machine, does not fully simulate the clinical procedure, it is the only possible option to guarantee the same standardized debonding procedure for debonding the brackets, which is not possible when brackets are removed by hand. Therefore, this procedure was chosen in the presented investigation to reduce possible confounders. In-vitro polishing is easier and not completely comparable to intraoral polishing as the access is easy and the specimens are plane, so the contact angle is different to tooth surfaces and there are no adjacent structures such as neighboring teeth. Further limitations are that the influence of polishing time was not investigated, neither was the impact of different operators. Therefore, further investigations are necessary here.

## Conclusion

Within the limitations of this study, the following conclusions can be drawn:

1. The higher the hardness values of the ceramic, the lower the surface roughness values. Therefore, ZrO2 and LiSi showed lower Ra values than LEU.
2. Soflex can be used for all investigated ceramic surfaces after all different pretreatments, but is only effective on rougher surfaces
3. Paste polishing cannot be recommended for the investigated ceramic/pretreatment combinations
4. The investigated Set should be used with caution and not on smooth surfaces.
5. Polishing after MEP on LiSi and LEU, and HF on LiSi surfaces might not be necessary.
6. None of the polishing methods seems to be recommendable for CoJet pretreated ZrO2

## Supplementary Information

Below is the link to the electronic supplementary material.

Supplementary file1 (DOCX 122 KB)

## Data Availability

The datasets generated during and/or analysed during the current study are available from the corresponding author on reasonable request.
