# Supplementary material for: Impact of polishing system on surface roughness of different ceramic surfaces after various pretreatments and bracket debonding
Source: Clin Oral Investig. 2023 May 11;27(8):4389–99. doi: 10.1007/s00784-023-05058-3 (PMC10415457; doi:10.1007/s00784-023-05058-3)
Supplement: Supplementary file 1 — Supplementary file1 (DOCX 122 KB) [file 784_2023_5058_MOESM1_ESM.docx]

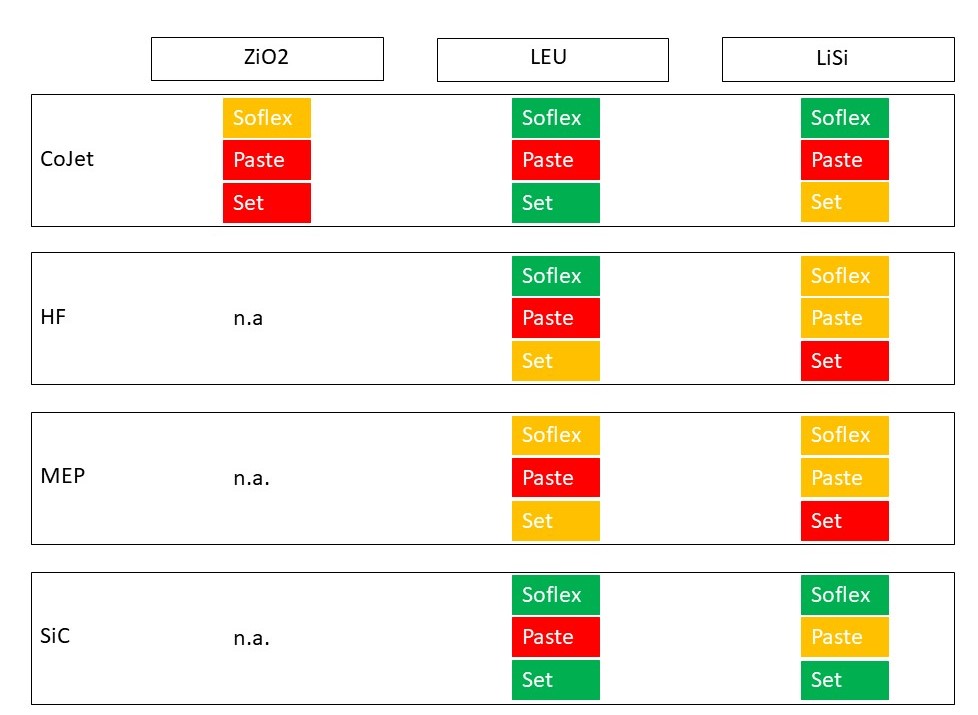


**Supplementary Figure 1: Recommended polishing procedures for each ceramic/pretreatment combination based on the Ra values before and after polishing.** Green = improved surface/ lower Ra value, (recommended), orange = no significant surface/Ra value change (moderately recommended), red = rougher surface/higher Ra value (not recommended); HF: hydrofluoric acid, MEP: Monobond Etch&Prime, SiC: silicium carbid grinder, ZiO2: zirconia, LEU:leucite, LiSi: lithium silicate, n.a. = not applicable
